# Integrating intra- and interpersonal perspectives on chronic low back pain: the role of emotion regulation and attachment insecurity

**DOI:** 10.3389/fpsyg.2024.1331227

**Published:** 2024-04-12

**Authors:** Yixin Yang, Dominik Mischkowski

**Affiliations:** ^1^Department of Psychology, Ohio University, Athens, OH, United States; ^2^Department of Psychology, University of Illinois at Urbana- Champaign, Champaign, IL, United States

**Keywords:** chronic low back pain, biopsychosocial model of pain, emotion regulation, attachment insecurity, psychosocial factors involved in chronic pain

## Abstract

**Objective:**

Chronic low back pain (CLBP) is burdensome and interferes with psychological and physical functioning of those affected. Past research has examined interpersonal (e.g., attachment insecurity) or intrapersonal factors (e.g., emotion regulation [ER]) involved in chronic pain. However, to enhance our understanding of CLBP’s biopsychosocial underpinnings, more empirical integration of both intra- and interpersonal factors involved in CLBP is needed. Thus, our study examined the independent and joint associations of insecure attachment dimensions and ER strategies with CLBP severity and interference.

**Methods:**

We recruited 242 US adults with CLBP through Prolific Academic, an online participant pool. Participants from Prolific Academic were eligible for the study if they were at least 18 years of age, resided in the US, reported CLBP at least half the days over the past 6 months (>3 months), and used prescribed pain medication for their CLBP. Data collection was between November 2021 and February 2022. Eligible participants filled out a Qualtrics survey which consisted of measures assessing insecure attachment dimensions, ER strategies, as well as demographical information. Outcome variables in the present study were CLBP severity and interference. We ran multiple linear regression models to examine the associations between ER strategies and insecure attachment dimensions as predictors, and CLBP severity or interference as predicted variables, after controlling for sex as a covariate; we also conducted moderation analyses to investigate the interactions between ER strategies and insecure attachment dimensions when testing associations with CLBP severity or interference.

**Results:**

Our results indicated that, after controlling for ER strategies, anxious attachment was positively associated with CLBP interference but not pain severity (*CI*: 0.101 to 0.569; *CI*: −0.149 to 0.186); avoidant attachment was not associated with CLBP interference or severity (*CI*: −0.047 to 0.511*; CI*: −0.143 to 0.256). After adjusting for anxious and avoidant attachment, emotional expression and expressive suppression were positively associated with CLBP severity (*CI*: 0.037 to 0.328; *CI*: 0.028 to 0.421) but not interference (*CI*: −0.003 to 0.403; *CI*: −0.406 to 0.143). Furthermore, emotional expression was associated with CLBP severity and interference at low and medium levels of avoidant attachment (*CI*: 0.165 to 0.682; *CI*: 0.098 to 0.455); expressive suppression and cognitive reappraisal did not interact with attachment dimensions when examining CLBP severity or interference (*CI*s: *LL*s ≤ −0.291 to *UL*s ≥ 0.030).

**Conclusion:**

Our study shows that anxious attachment may be an interpersonal risk factor related to CLBP, above and beyond intrapersonal ERs, as anxious attachment was associated with higher levels of pain interference. Furthermore, emotional expression was associated with increased CLBP severity and interference, particularly among individuals at low and medium levels of avoidant attachment. Existing studies on chronic pain have mostly focused on examining intrapersonal or interpersonal correlates in isolation. The present study extends our understanding of CLBP by considering the role of interpersonal factors (i.e., insecure attachment dimensions), in combination with intrapersonal ER strategies. Given the correlational nature of the present study, longitudinal studies are needed to establish causality between psychosocial correlates and CLBP symptoms. Ultimately, we hope our integrated approach will facilitate the development of treatments and interventions tailored to address patients’ attachment-related needs, enhancing the management and maintenance of CLBP among patients.

## Introduction

Chronic low back pain (CLBP) is a chronic pain condition with many adverse consequences for those afflicted, including disability (Vos et al., 2017) and poor psychological well-being (Baird and Sheffield, 2016). The causes of CLBP are multi-dimensional, with a complex interplay between biological (Chan et al., 2012), psychological (Sagheer et al., 2013), and social factors (Fransen et al., 2002). While some chronic pain conditions have biomechanical causes (Pope et al., 1985; Shin and Mirka, 2007), a large body of research also documents psychological factors underlying chronic pain, such as dysfunctional emotion regulation (ER) processes (Geenen et al., 2012). Specifically, expressive suppression is an ER strategy generally considered maladaptive as individuals suppress the expression of negative emotions (Gross and John, 2003), which may exacerbate chronic pain symptoms (van Middendorp et al., 2008). Conversely, cognitive reappraisal, which indicates individuals’ effort to reframe a stressor as less threatening (Gross and John, 2003), may reduce chronic pain symptoms (Seminowicz et al., 2013). Another ER is emotional expression, which refers to disclosing inner thoughts verbally or in a written format (Stanton et al., 2000). Emotion expression has also been related with better chronic pain outcomes (Geenen et al., 2012).

Despite the theoretical linkage between ER and chronic pain symptoms, research on the association between ER and chronic pain has produced inconsistent findings. For instance, some researchers did not find connections of ER with chronic pain intensity or disability (Wong and Fielding, 2013), while other researchers found that different ER strategies are associated with chronic pain symptoms only in subsets of chronic pain patients (Geenen et al., 2012). One possible explanation for these inconsistencies is that most of these studies have focused on ER as an *intra*personal process, while chronic pain management usually happens in an *inter*personal, social context (Pietromonaco et al., 2013). Accordingly, some researchers have begun focusing on interpersonal correlates of chronic pain, such as adult attachment dimensions (Meredith et al., 2008).

According to Hazan and Shaver (1987), the core tenet of adult attachment theory is that interpersonal interactions with significant others influence an individual’s mental schemas of the self and others. These mental schemas guide an individual’s behavior when seeking out their romantic partner, to obtain closeness and security as a response to stressful situations (Hazan and Shaver, 1987; Fraley and Shaver, 2000). Anxious attachment and avoidant attachment are two insecure attachment dimensions underlying adult attachment insecurity (Hazan and Shaver, 1987). Individuals high in avoidant attachment are conceptualized as having difficulty trusting intimacy (Collins and Allard, 2001; Collins and Gillath, 2012) and as avoiding emotional closeness due to an excessive need for independence in romantic relationships (Collins and Gillath, 2012). Unlike avoidant individuals, individuals high in anxious attachment are described as having low self-regard, desiring excessive intimacy and closeness, and being overly sensitive to cues of rejection and abandonment from their romantic partner (Collins and Allard, 2001). Conversely, individuals who score low on both avoidant and anxious dimensions are classified as having secure attachment. Securely attached individuals appear to be more comfortable with intimacy and closeness and believe that their romantic partner will be available and dependable during times of threat (Shaver and Mikulincer, 2006).

There have been several empirical studies postulating a linkage between attachment insecurity and chronic pain. Generally, compared to securely attached patients with chronic pain, those who are insecurely attached tend to experience more adverse pain outcomes, such as increased disability, greater pain intensity (McWilliams et al., 2000), elevated psychological distress, such as anxiety (Meredith et al., 2005), pain catastrophizing, and depression (Mikulincer and Florian, 1998; Ciechanowski et al., 2003). Despite the established linkage between attachment insecurity and chronic pain found in the literature, several empirical gaps need to be filled.

First and foremost, it remains unclear whether attachment insecurity is simply an interpersonally regulated type of maladaptive ER, or whether it is associated with chronic pain symptoms above and beyond strategies more traditionally considered as ER. Second, existing theoretical models that explore the complex interplay between intra- (i.e., ER strategies) and interpersonal factors (i.e., adult attachment dimensions) are still very rare, with most of them focusing on independent predictors linked to chronic pain solely (e.g., Anderson and Hines, 1994; Mikail et al., 1994), overlooking the interactive associations of interpersonal and intrapersonal factors that may underlie chronic pain. Notably, a considerable number of CLBP cases are not caused by physical injuries, but rather categorized as non-specific, that is, unclear in their origins (Deyo et al., 2015; Maher et al., 2017). This may suggest a potential interplay of intrapersonal and interpersonal processes in shaping CLBP experiences, as both intra- and interpersonal factors have been found to be associated with the development and maintenance of chronic pain conditions (McWilliams et al., 2000; van Middendorp et al., 2008; Seminowicz et al., 2013). Thus, we believe it is important to test the involvement of these interpersonal (i.e., insecure attachment dimensions) and intrapersonal factors (i.e., ER strategies) related to CLBP not separately but rather, *integrally*, because the combined effects may account for additional variability in individuals’ CLBP symptoms.

Evidence from existing theoretical frameworks and studies in attachment insecurity and chronic pain literature supports this notion. Meredith et al. (2008) suggested that individuals with chronic pain may engage in various emotional/behavioral regulations to cope with the pain, depending on their standing on attachment dimensions. Notably, individuals with high avoidant attachment tend to exhibit less expression of emotions and more pain denial as coping mechanisms for chronic pain (Kotler et al., 1994; Schmidt et al., 2002). Such coping pattern may, in turn, be associated with unfavorable outcomes in patients with chronic pain due to less support-seeking behaviors and a subsequent decrease in attention and caregiving from significant others (Matire et al., 2002; Romeo et al., 2017). As suggested by studies on dyadic coping of chronic pain, significant others are largely considered the primary caregivers and a resource of emotional support for patients with chronic pain (Prenevost and Reme, 2017; Mittinty et al., 2020). Research indicates that individuals who receive negative responses from their partners, as opposed to those with supportive partners, tend to suffer from more pain symptoms, including greater pain-related activity interference and more depressive symptom severity (Stroud et al., 2006).

The present paper takes a step toward resolving the ambiguity in the chronic pain literature by considering both the *unique* and *joint* influences of insecure attachment dimensions and ER strategies among a group of CLBP patients. The goal of the present research was thus twofold: Firstly, we aimed to show that insecure attachment dimensions (i.e., anxious attachment, avoidant attachment) are associated with CLBP severity or interference, above and beyond ER variables. Specifically, we hypothesized that anxious attachment and avoidant attachment may be associated with higher CLBP severity or interference, after controlling for ER strategies. Furthermore, it is unclear whether the associations between ERs and CLBP may be increased or attenuated depending on the person’s attachment dimension. Therefore, we tested whether ERs interact with attachment dimensions in their associations with CLBP severity or interference. We hypothesized that associations between ER strategies and CLBP severity or interference may depend on where individuals fall on anxious or avoidant attachment dimensions.

**Figure 1:**
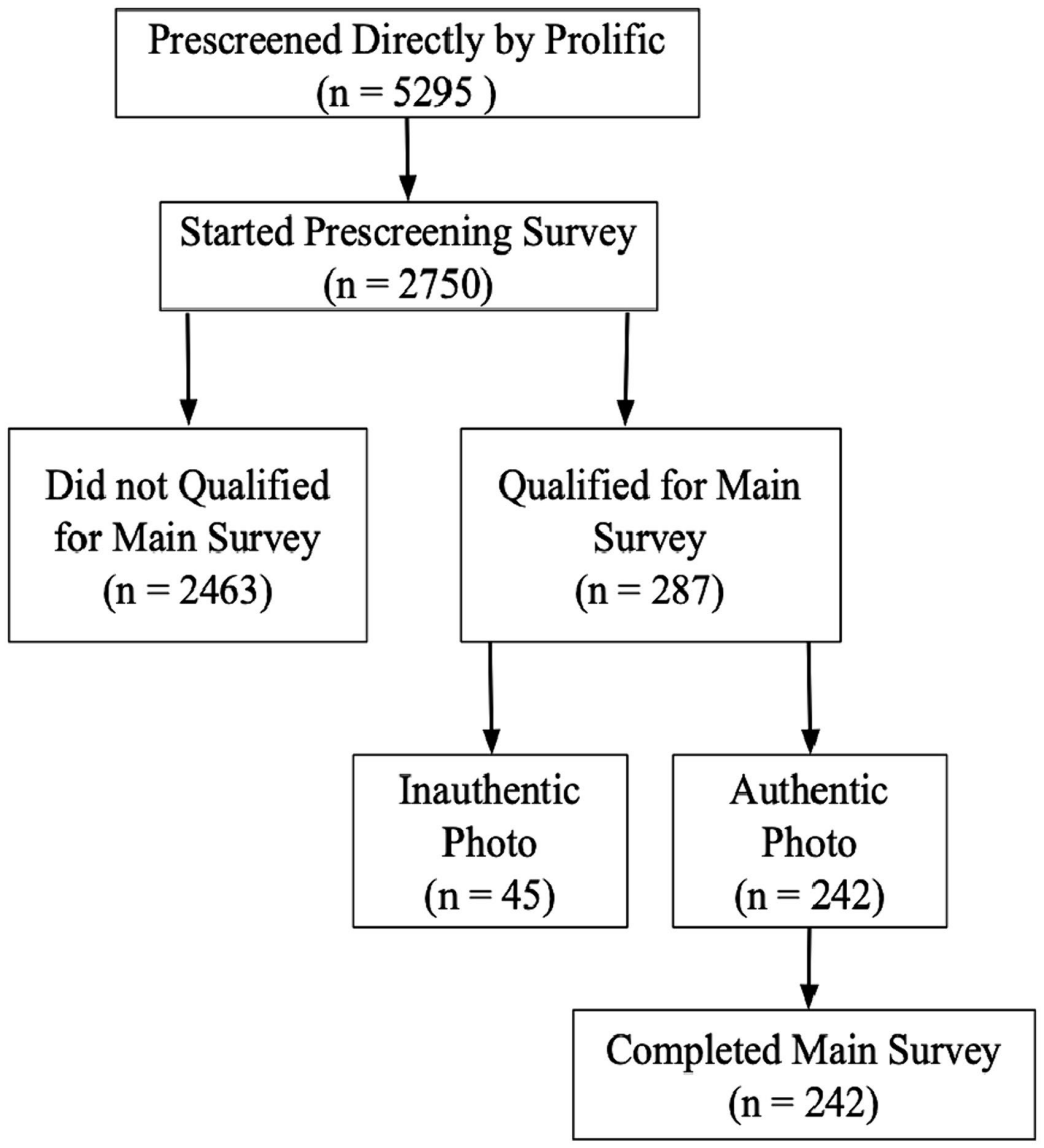
Flowchart of participation in this study.

We conducted a cross-sectional study to investigate the correlates of CLBP severity and interference from a psychosocial perspective. Specifically, we recruited online participants with CLBP who had been prescribed medication to relieve their pain, using an established photo validation procedure (Ysidron et al., 2022). Prospective participants were invited to upload a photo of their prescribed pain medication(s) with a handwritten time stamp included in the photo indicating the date on which the photo was taken (Ysidron et al., 2022). The handwritten time stamp was intended to ensure that the medication picture uploaded by participants was not fake (e.g., a photo downloaded online). By so doing, we ensured that our online participants indeed were suffering from CLBP instead of falsifying their medical status to gain monetary benefits (for the details of our study procedure, i.e., the number of malingering participants, see Figure 1).

## Materials and methods

### Participants

We recruited 242 CLBP participants from Prolific Academic (*M*_age_ = 36.9, *SD* = 10.1). Our sample included 71.5% females and 28.1% males; 74.0% of the participants were Caucasian Americans, 14.0% African Americans, 2.9% Asians/Asian Americans, 0.8% American Indian/Alaskan Native, 0.4% Native Hawaiian/Other Pacific Islander, and 7.4% being other race; 93.0% of the participants were non-Hispanic, and 6.6% were Hispanic/Latino; 0.4% of the participants did not provide demographic information such as age and gender. Participants from Prolific Academic were eligible for the study if they were at least 18 years of age, resided in the US, had CLBP at least half the days over the past 6 months (>3 months; Andersson, 1999; Deyo et al., 2015), and used prescribed pain medication for their CLBP. We collected data between November 2021 and February 2022.

#### Power analysis

We conducted a power analysis using G*Power based on an unpublished pilot study conducted among participants with mostly *acute* (not chronic) low back pain. With a significance criterion of *α* = 0.05 and power = 0.80, results suggested that a sample size of at most 237 participants was enough to replicate significant associations (*r*s ≥ 0.18) between anxious or avoidant attachment and CLBP severity or interference found in the pilot study.

#### Survey procedures

To ensure that our sample consisted of people with CLBP, we ran two separate surveys through Prolific Academic: An initial, short survey and a longer, main survey. See Figure 1 for a recruitment flowchart of this study. Using the respective prescreening criteria applied by Prolific Academic, we made the initial survey accessible only to prospective participants who were 18 years and older, resided in the US, and had *any* chronic pain symptoms (eligible *N* = 5,295). Eligible participants who agreed to participate in the short survey were asked to indicate whether they had CLBP, *specifically (yes/no)*, and whether they took prescribed medication to relieve CLBP symptoms *(yes/no)*. Those who claimed to have CLBP and took prescribed pain medication were invited to upload a photo of their pain medication(s) with a handwritten note included in the photo indicating the date at which the photo was taken (Ysidron et al., 2022). We also instructed participants to make sure the medication label in the photo was readable so that we could clearly verify that the prescription constituted pain medication. The first author of this study and a trained research assistant coded photos (with identifiers removed from the picture) as authentic or not. We invited in the second, main survey only participants who submitted authentic photos of prescribed pain medication to participate. This second survey contained measures of attachment dimensions, ER strategies, CLBP severity and interference, as well as demographics. We did not invite participants to the main survey if they did not upload a photo or uploaded a photo which at least one coder categorized as unauthentic (*n* = 45, see the flowchart in Figure 1 for details). The Ohio University Institutional Review Board approved all study procedures.

#### Measures

#### Attachment anxiety and avoidance

We measured attachment anxiety and avoidance using the respective subscales of the Experiences in Close Relationship Scale – Revised (ECR-R; Brennan et al., 1998). The ECR-R is an established 36-item questionnaire assessing two types of insecure adult attachment styles, including anxious attachment and avoidant attachment in romantic relationships. In our study, the ECR-R showed strong internal consistency (anxious attachment Cronbach’s *α* = 0.95; avoidant attachment Cronbach’s *α* = 0.95), which is consistent with existing literature on the scale’s psychometric properties (Sibley and Liu, 2004; Busonera et al., 2014).

#### Cognitive reappraisal and expressive suppression

To measure CLBP-related cognitive reappraisal and expressive suppression, we administered a modified version of the Emotion Regulation Questionnaire (ERQ; Gross and John, 2003). The ERQ is an established 10-item scale assessing an individual’s tendency to rely on cognitive reappraisal and expressive suppression when regulating negative emotions. Because the original ERQ was not designed to measure how individuals cope with negative emotions related to chronic pain, we reworded the ERQ to make items CLBP-specific. Participants thus responded to items such as “I control my emotions about my CLBP by changing the way I think about the situation I am in,” “When my CLBP puts me in a stressful situation, I make myself think about it in a way that helps me stay calm,” “I keep my emotions regarding my CLBP to myself,” or “I control my emotions regarding my CLBP by not expressing them” on 7-point Likert-type scales ranging from 1 (*strongly disagree*) to 7 (*strongly agree*). The ERQ has demonstrated good criterion and incremental validity and internal consistency in general community samples (Ioannidis and Siegling, 2015; Preece et al., 2019). In our study, both the cognitive reappraisal (Cronbach’s *α* = 0.91) and expressive suppression (Cronbach’s *α* = 0.76) subscales showed acceptable internal consistency.

#### Emotion expression

We measured CLBP-related emotional expression as another common ER strategy. Specifically, we modified two items derived from Cameron and Overall (2018)’s emotional expression measure to make these items CLBP-specific. Participants responded to the items: “I shared and discussed my thoughts and feelings about my CLBP” and “I expressed my true emotions about my CLBP” on 7-point Likert scales ranging from 1 (*strongly disagree*) to 7 (*strongly agree*). Items were internally consistent (Cronbach’s *α*  = 0.94) in our study.

#### CLBP severity and interference

Participants rated their CLBP on the pain severity and pain interference subscales of the Brief Pain Inventory (BPI) – Short Form (Cleeland and Ryan, 1991). The BPI-SF has demonstrated strong reliability, in addition to construct, convergent, and predictive validity (Mendoza et al., 2006). The pain severity subscale assesses the intensity of CLBP “right now,” “on average,” “at its worst during the last 24 h,” and “at its least during the last 24 h.” Participants responded on 11-point Likert scales, ranging from 0 (*no pain*) to 10 (*pain as bad as you can imagine*). The pain interference subscale assessed the debilitating effects of CLBP in people’s daily lives and covered seven domains: general activity, mood, walking ability, normal work (both work outside the home and housework), relations with other people, sleep, and enjoyment of life. Participants indicated the extent to which CLBP had interfered during the last 24 h with these seven domains on 11-point Likert scales, ranging from 0 (*does not interfere*) to 10 (*completely interferes*). Items were internally consistent, both for the pain severity (Cronbach’s *α* = 0.85) and interference (Cronbach’s *α* = 0.92) subscales.

### Participant characteristics

Participants completed a series of questions about their sex, age, race, and ethnicity.

### Statistical analysis

Data were analyzed using SPSS, version 28 (IBM Corp, 2021), and Macro PROCESS (Hayes, 2012). We calculated zero-order correlations among all study variables. We summed participants’ responses to all study variables, such that higher scores indicates higher endorsement of the study variable. Next, we conducted multiple linear regressions to examine the unique and interactive associations of insecure adult attachment dimensions and ER strategies with CLBP severity or pain interference. When testing whether ER strategies interacted with insecure attachment dimensions when testing associations with CLBP severity and interference, we mean-centered ER and attachment variables, so we could interpret main effects and intercepts at regressor means. In these analyses, we used Model 15 of the Macro PROCESS (Hayes, 2012) to account for the substantial correlation between avoidant and anxious attachment (*r* = 0.55, *p* < 0.001, see Table 1). We tested the interaction between a specific ER variable (e.g., emotional expression) and a specific attachment variable (e.g., avoidant attachment), while not only controlling for the other attachment variable (e.g., anxious attachment) but also for the interaction term of the ER variable and the other attachment variable (e.g., the emotional expression X anxious attachment interaction), as it is necessary when controlling for a third variable in interaction analyses (Muller et al., 2005). All tests for significance were two-sided, and *p*-values smaller than 0.05 were considered statistically significant. We restricted analyses to participants who completed the entire survey.

**Table 1:** Zero-order correlations between all predictor variables and outcome variables in the main study.

Variables	n	M	SD	1	2	3	4	5	6	7	8
1. Anxious attachment	242	60.71	26.21	–							
2. Avoidant attachment	242	56.00	23.84	0.55^***^	–						
3. Expressive suppression	242	28.27	7.81	0.21^***^	0.31^***^	–					
4. Cognitive reappraisal	242	16.51	5.22	−0.16^*^	−0.07	0.18^*^	–				
5. Emotional expression	241	9.63	3.57	−0.21^***^	−0.43^***^	−0.54^***^	0.08	–			
6. Pain severity	241	21.91	6.46	0.05	0.05	0.05	0.01	0.09	–		
7. Pain interference	241	41.05	16.19	0.24^***^	0.17^*^	−0.09	−0.04	0.10	0.62^***^	–	
8. Sex	241	–	–	−0.01	0.11	−0.18^***^	0.07	0.09	0.17^***^	0.14^**^	–

*p < 0.05; **p < 0.01; ***p < 0.001.

## Results

### Zero-order correlations between ER, attachment, and CLBP severity or interference

For zero-order correlations between all study variables (see Table 1). Both anxious attachment and avoidant attachment were positively correlated with CLBP interference but not with CLBP severity. Expressive suppression, cognitive appraisal, or emotional expression did not correlate with either CLBP severity or interference.

### Unique associations of insecure attachment and ER with CLBP severity or interference

#### Attachment

We postulated that anxious attachment or avoidant attachment should be positively associated with CLBP severity or interference, above and beyond ER variables. To test this hypothesis, we entered anxious attachment, avoidant attachment, expressive suppression, cognitive reappraisal, emotional expression, and sex into two regression models with CLBP severity (*F*(6,234) = 2.510, *p* = 0.023, *R*^2^ = 0.06) or pain interference (*F*(6,234) = 4.734, *p* < 0.001, *R*^2^ = 0.11) as regressands. We included sex as a covariate in these analyses because sex significantly correlated with both pain severity (*F*(1,239) = 6.868, *p* = 0.009, *d* = 0.08) and pain interference (*F*(1,239) = 4.465, *p* = 0.036, *d* = 0.07). See Tables 2, 3 for full regression models.

**Table 2:** Regression results using pain severity as the criterion.

Variables	Unstandardized coefficient	SE	Standardized coefficient	*t*	*p*	B	Beta
(Constant)	2.570	0.859		2.992	0.003
Anxious attachment	0.019	0.085	0.017	0.221	0.825
Avoidant attachment	0.057	0.101	0.047	0.560	0.576
Expressive suppression	0.224	0.100	0.182	2.246	0.026
Cognitive reappraisal	−0.052	0.084	−0.042	−0.616	0.538
Emotional expression	0.183	0.074	0.202	2.476	0.014
Sex	0.643	0.236	0.180	2.721	0.007

Dependent variable: pain severity. *F*(6,234) = 2.510, *p* = 0.023, *R*^2^ = 0.06.

**Table 3:** Regression results using pain interference as the criterion.

Variables	Unstandardized coefficient	SE	Standardized coefficient	*t*	*p*	B	Beta
(Constant)	2.750	1.200		2.292	0.023
Anxious attachment	0.335	0.119	0.212	2.823	0.005
Avoidant attachment	0.232	0.142	0.133	1.638	0.103
Expressive suppression	−0.131	0.139	−0.074	−0.941	0.348
Cognitive reappraisal	−0.003	0.117	−0.001	−0.022	0.982
Emotional expression	0.200	0.103	0.155	1.945	0.053
Sex	0.493	0.330	0.096	1.496	0.136

Dependent variable: pain interference. *F*(6,234) = 4.734, *p* < 0.001, *R*^2^ = 0.11.

After controlling for all other regressors, anxious attachment was positively associated with CLBP interference but not CLBP severity (*B* = 0.335, 95% *CI*: 0.101 to 0.569, *β* = 0.212, *t* = 2.823, *p* = 0.005; *B* = 0.019, 95% *CI*: −0.149 to 0.186, *β* = 0.017, *t* = 0.221, *p* = 0.825). In contrast, avoidant attachment was not associated with CLBP interference or CLBP severity (*B =* 0.232, 95*% CI*: −0.047 to 0.511, *β =* 0.133, *t =* 1.638, *p =* 0.103*; B =* 0.057, 95% *CI*: −0.143 to 0.256, *β =* 0.047, *t =* 0.560, *p =* 0.576), after controlling for all other regressors.

#### Emotion regulation

Furthermore, in these regression models, expressive suppression was significantly associated with pain severity but not pain interference (*B* = 0.224, 95% *CI*: 0.028 to 0.421, *β* = 0.182, *t* = 2.246, *p* = 0.026; *B* = −0.131, 95% *CI*: −0.406 to 0.143, *β* = −0.074, *t* = −0.941, *p* = 0.348). Moreover, emotional expression was positively associated with pain severity but was not significantly related to pain interference (*B* = 0.183, 95% *CI*: 0.037 to 0.328, *β* = 0.202, *t* = 2.479, *p* = 0.014; *B* = 0.200, 95% *CI*: −0.003 to 0.403, *β* = 0.155, *t* = 1.945, *p* = 0.053). Cognitive reappraisal was not significantly associated with either pain severity or pain interference, after controlling for other predictors (*B* = −0.052, 95% *CI*: −0.217 to 0.114, *β* = −0.042, *t* = − 0.616, *p* = 0.538; *B* = −0.003, 95% *CI*: −0.234 to 0.228, *β* = −0.001, *t* = −0.022, *p* = 0.982). Sex was significantly associated with pain severity but not pain interference (*B* = 0.643, 95% *CI*: 0.177 to 1.108, *β* = 0.180, *t* = 2.721, *p* = 0.007; *B* = 0.493, 95% *CI*: −0.156 to 1.143, *β* = 0.096, *t* = 1.496, *p* = 0.136), after controlling for attachment dimensions and ER strategies in the regression model.

#### Interactive associations of emotion regulation and attachment with CLBP severity or interference

We also examined whether ER strategies (i.e., emotional suppression, cognitive reappraisal, and emotional expression) interacted with insecure attachment dimensions (e.g., anxious attachment or avoidant attachment) when testing associations with CLBP severity and interference.

#### Emotional expression and avoidant attachment interacted when testing associations with CLBP severity

**Figure 2:**
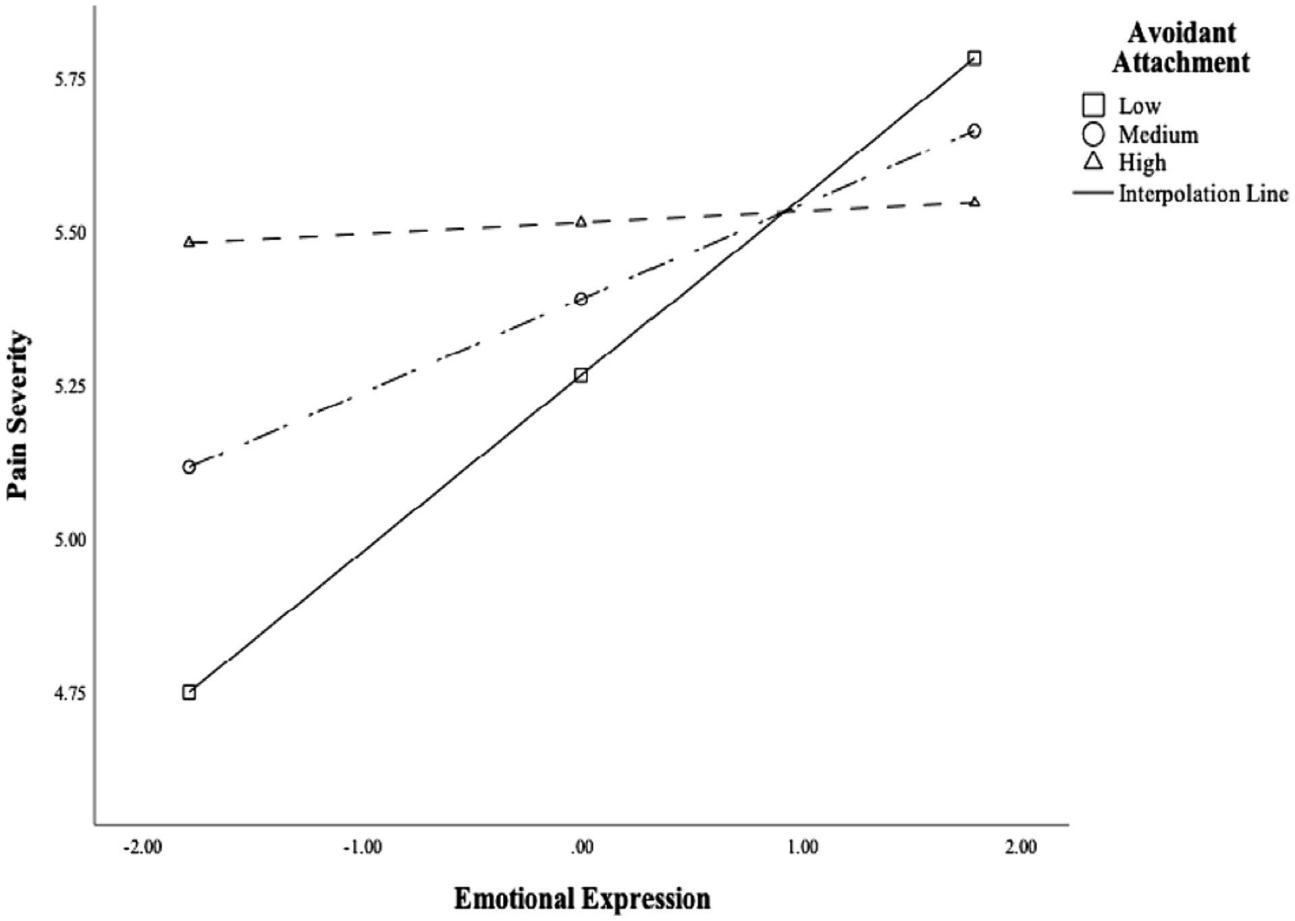
Emotional expression interacted with avoidant attachment when testing the association with chronic low back pain severity.

We regressed CLBP severity on emotional expression, avoidant attachment, the emotional expression X avoidant attachment interaction, while also controlling for anxious attachment, sex, and the interaction term between anxious attachment and emotional expression. Emotional expression interacted with avoidant attachment when testing associations with CLBP severity [*F*(2,238) = 50.996, *p* < 0.001, *R*^2^ = 0.30; *B* = −0.114, *CI*: −0.207 to −0.020, *SE* = 0.048, *t* = −2.396, *p* = 0.017, for a depiction of this interaction, see Figure 2].

To probe the association between emotional expression and CLBP severity at different levels of avoidant attachment, we conducted simple slope analyses. Specifically, at low (1 *SD* below the mean) and medium (mean) levels of avoidant attachment, emotional expression was positively related to CLBP severity (−1 *SD*: *B* = 0.423, *CI*: 0.165 to 0.682, *SE* = 0.131, *t* = 3.228, *p* = 0.001; mean: *B* = 0.277, *CI*: 0.098 to 0.455, *SE* = 0.091, *t* = 3.049, *p* = 0.003). At high levels of avoidant attachment (1 *SD* above the mean), emotional expression was not related to CLBP severity (*B =* 0.130, *CI*: −0.084 to 0.344, *SE =* 0.109, *t =* 1.194, *p =* 0.234). These analyses suggested that expressing more CLBP-related emotions was associated with *greater* pain severity among people with low or moderate, but not high, levels of avoidant attachment. Moreover, people with low emotional expression and low levels of avoidant attachment seemed to have the lowest levels of pain severity, suggesting that this combination may be protective.

Importantly, emotion expression was substantially correlated with expressive suppression (*r* = −0.54, *p* < 0.001, see Table 1). To control for any confounding effects of expressive suppression, we added expressive suppression and the two-way interaction term between expressive suppression and avoidant attachment into the regression model and reran the analysis. The interaction between emotional expression and avoidant attachment when testing associations with CLBP severity remained robust even after controlling for expressive suppression and the two-way interaction between expressive suppression and avoidant attachment (*F*(2,238) = 50.996, *p* < 0.001, *R*^2^ = 0.30; *B* = −0.100, *CI*: −0.193 to −0.002, *SE* = 0.049, *t* = −2.017, *p* = 0.044).

#### Emotional expression and avoidant attachment interacted when testing associations with CLBP interference

**Figure 3:**
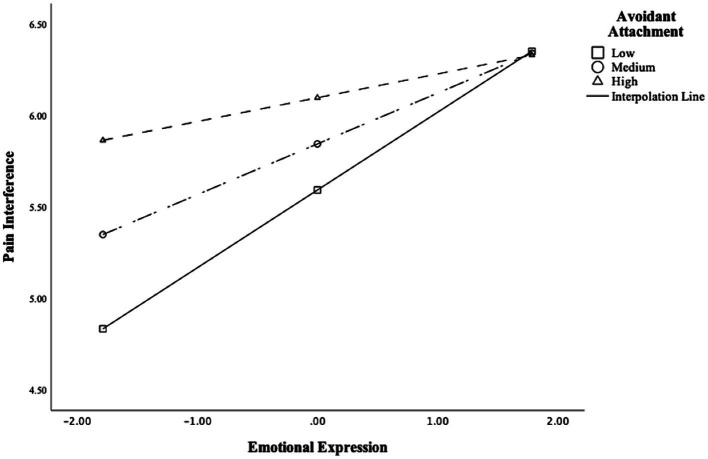
Emotional expression interacted with avoidant attachment when testing the association with chronic low back pain interference.

After controlling for sex, anxious attachment, and the two-way interaction term between anxious attachment and emotional expression, emotional expression interacted with avoidant attachment when testing associations with CLBP interference [*F*(2,238) = 50.996, *p* < 0.001, *R*^2^ = 0.30; *B* = −0.133, *CI*: −0.263 to −0.003, *SE* = 0.066, *t* = −2.013, *p* = 0.045, for a graphical depiction of this interaction, see Figure 3]. Next, we conducted simple slope analysis to probe the significant interaction at different levels of avoidant attachment (−1 *SD*, mean, +1 *SD*). Again, at low (1 *SD* below the mean) and medium levels of avoidant attachment, emotional expression was significantly and positively associated with pain interference (−1 *SD*: *B* = 0.423, *CI*: 0.165 to 0.682, *SE* = 0.131, *t* = 3.228, *p* = 0.001; mean: *B* = 0.277, *CI*: 0.098 to 0.455, *SE* = 0.091, *t* = 3.049, *p* = 0.003). At high (1 *SD* above the mean) level of avoidant attachment, emotional expression did not relate to pain interference (*B* = 0.130, *CI*: −0.084 to 0.344, *SE* = 0.109, *t* = 1.194, *p* = 0.234).

Again, because emotional expression was correlated with expressive suppression, we added expressive suppression and the two-way interaction term between expressive suppression and avoidant attachment into the regression model and reran the analysis. After controlling for expressive suppression and the two-way interaction term expressive suppression and avoidant attachment, emotional expression no longer moderated the association between avoidant attachment and pain interference (*F*(2,238) = 50.996, *p* < 0.001, *R*^2^ = 0.30; *B* = −0.123, *CI*: −0.258 to 0.012, *SE* = 0.068, *t* = −1.797, *p* = 0.074).

#### No other interactions between ER strategies and insecure attachment dimensions when testing associations with CLBP severity or interference

Emotional expression did not interact with anxious attachment when testing the associations with pain severity or pain interference, after controlling for sex, avoidant attachment, and the two-way interaction term between avoidant attachment and emotional expression. Similarly, expressive suppression and cognitive reappraisal did not interact with either anxious attachment or avoidant attachment when testing associations with CLBP severity or interference (all |*B*s| ≤ 0.131, *SE*s ≥ 0.081, |*t*s| ≤ 1.605, *p*s ≥ 0.110, *CI*s: *LL*s ≤ −0.291 to *UL*s ≥ 0.030).

## Discussion

Our study suggested that anxious attachment was positively associated with CLBP interference, above and beyond ER strategies. Our finding is consistent with extant literature showing that anxiously attached individuals tend to suffer from more CLBP-related functional impairments. Several explanations may account for this finding. First, existing studies suggest that people characterized by anxious attachment may have inadequate psychosocial resources to cope with chronic pain conditions (Anderson and Hines, 1994; Meredith et al., 2008). In addition, the increased burden of CLBP on anxiously attached patients may increase their dependence for support upon significant others (Suso-Ribera et al., 2020). This idea is consistent with the finding that having anxious attachment relative to secure attachment is associated with lower pain self-efficacy, that is, a tendency for patients to underestimate their own capacity to cope with pain and to fulfill daily commitments and activities while experiencing pain (Meredith et al., 2006; Nicholas, 2007). By focusing on insecure attachment dimensions after controlling for intrapersonal ER strategies, our work suggests an important role for anxious attachment in the CLBP experience, given that insecure interpersonal dynamics between CLBP patients and their significant others may be associated with functional impairments caused by CLBP. Thus, we believe our work contributes to the current literature on adult attachment factors underlying CLBP, explaining unique variance in CLBP interference above and beyond intrapersonal ER strategies.

After adjusting for ER variables (and sex as a covariate), anxious attachment was not associated with pain severity. It is important to acknowledge that existing literature on the association between attachment dimensions and chronic pain symptoms is quite mixed. Despite the lack of significant associations between insecure attachment dimensions and CLBP severity, our findings are consistent with other studies, in which insecure attachment was not associated with higher chronic pain intensity (Ciechanowski et al., 2003; McWilliams, 2017; Peñacoba et al., 2018), Thus, more research is needed to further explore the associations of anxious attachment with CLBP severity.

Similarly, avoidant attachment was not associated with CLBP severity or interference when controlling for ER variables (and sex). The null association between avoidant attachment and chronic pain severity has also been reported previously (e.g., Andersen et al., 2011). The reasons for these non-significant associations in the present study were somewhat unclear. However, at least zero-order correlations suggested that avoidant attachment was significantly and positively associated with pain interference. It is plausible that, after we controlled for covariance between anxious attachment and avoidant attachment (*r* = 0.55, *p* < 0.001) and, possibly, for an underlying, higher-order, attachment insecurity dimension, in our multiple regression models, the significant zero-order correlation between avoidant attachment and pain interference disappeared.

Though only the secondary focus of this research, both emotional expression and expressive suppression were uniquely and positively associated with CLBP severity but not interference. Interestingly, our finding on the positive association between emotional expression and CLBP severity seems to indicate that overly expressing CLBP-related distress is maladaptive, as emotional expression – especially distress expression – does not always have health benefits (Kennedy-Moore and Watson, 2001; Burns et al., 2015a,b). Our finding is consistent with ER research showing that the expression of pain-related negative emotions, such as anger, is associated with heightened pain severity among individuals with CLBP (Burns et al., 2015a,b). Importantly, CLBP patient’s emotional expressions can influence their significant others (Burns et al., 2015a,b; Weitkamp et al., 2021). It is thus possible that CLBP patients who tend to excessively and repeatedly share their pain-related negative emotions and catastrophize their pain may perceive more criticism and hostility from their spouse and significant others (Burns et al., 2015a,b). This, in turn, may be associated with an increased pain severity over time (Burns et al., 2013). Conversely, substantial research has suggested that *suppressing* negative emotions may also worsen chronic pain symptoms (e.g., Burns et al., 2008). Taken together, our results imply the possibility that both overly suppressing and sharing negative emotions constitute maladaptive ER strategies in the context of CLBP, correlating with more adverse outcomes, including exacerbated CLBP severity.

In addition to the statistically significant independent associations of anxious attachment and emotional expression with CLBP, analyses probing the interaction between ERs and insecure attachment dimensions showed that emotional expression may be associated with more pain among people with *low* and *medium* levels of avoidant attachment. Notably, emotional expression interacted with avoidant attachment when testing the association with CLBP severity (but not interference), even after controlling for expressive suppression which correlated moderately with emotional expression. This finding suggests that the interaction of emotional expression with avoidant attachment does not just reflect a lack of emotional suppression. In contrast, among highly avoidant people, there was no difference observed in CLBP severity or interference as a function of how much participants expressed CLBP-related emotions. These results may suggest that being avoidantly attached is already a risk factor for moderate to high levels of CLBP, so the disclosure of pain-related emotions no longer adds to the severity of CLBP symptoms among people high in avoidant attachment.

### Theoretical and practical implications

Our study contributes to the extant literature on insecure attachment, ER, and CLBP in several ways. Although CLBP does have biological causes underlying its occurrence (Chan et al., 2012), the present study focuses on CLBP from a psychosocial perspective, providing additional evidence for the associations of intra- and interpersonal factors, specifically insecure attachment dimensions, with CLBP severity and interference. Our study extended previous research by showing that anxious attachment explains significant variance in the pain experience among CLBP patients, above and beyond intrapersonal ER strategies, such that people with higher anxious attachment might experience more pain interference as a consequence of suffering from CLBP.

Moreover, our findings also contribute to previous research on the role of emotional expression and expressive suppression in CLBP. The present study is consistent with several existing findings on the negative health outcomes of expressing emotions (e.g., Broderick et al., 2005; Geenen et al., 2012) by showing that emotional expression may be associated with maladaptive CLBP outcome, such as increased pain severity. Relatedly, our study also examined the interactions between ER strategies and insecure attachment dimensions to more fully explain pain variance observed in a CLBP population. Specifically, results suggested that the association of emotional expression with CLBP severity may depend upon the specific attachment type and level of an individual: Emotional expression was associated with increased CLBP severity and interference at low and medium levels of avoidant attachment, but this pattern of associations was not observed among people with high avoidant attachment. In summary, these results suggested that disclosing worries related to CLBP may not be necessarily related to better pain symptoms, and that associations between (intrapersonal) emotion expression and CLBP may depend on interpersonal factors, such as having an avoidant attachment orientation.

Notably, some acute pain studies have embraced a similar integrated approach that takes into account the combined effects of various psychosocial and demographic factors to understand acute pain experiences among healthy subjects. For instance, studies using the cold pressor task (CPT) have examined how cognitive, psychological attributes (e.g., pain catastrophizing, pain self-efficacy; Diotaiuti et al., 2021) or demographic characteristics (i.e., gender; Diotaiuti et al., 2022) influence individuals’ perceptions of experimentally-induced acute pain. The present study contributes to this body of research by testing the interaction effects of ER strategies and insecure attachment dimensions in a sample of *chronic* LBP patients. Ultimately, we hope our integrated approach which combines both intra- and interpersonal psychosocial factors to understand CLBP will inform new clinical interventions aimed at the development of more personalized treatment that addresses not only the physical symptoms but also intrapersonal (e.g., ER coping strategies) and interpersonal factors (e.g., attachment dimensions) that may influence CLBP experiences.

### Limitations

There are some limitations of the present study, and some of these limitations may undermine the generalizability of our findings to other CLBP populations. Firstly, the cross-sectional nature of our data limits causal conclusions regarding the associations between insecure attachment dimensions, ER strategies, and CLBP symptoms. Second, because our study used participants’ medication prescriptions as a required prescreening criterion to verify the pain status of our subjects, our findings are based on a sample that mainly consisted of individuals undergoing pharmacological treatment for CLBP. Therefore, results from this study may not be applicable to CLBP patients who (1) do not seek pharmacological medical care or (2) rely on over-the-counter (OTC) medications rather than prescribed medications to alleviate their CLBP symptoms. In addition, the photo verification procedure (Ysidron et al., 2022) adopted in the study to prevent malingering individuals from participating may have inadvertently impacted the representativeness of our CLBP sample for various reasons. Notably, certain participants might have been excluded from our research if they either (1) chose not to upload a photo of their medication due to privacy concerns, (2) employed alternative medical interventions, or (3) did not have immediate access to their medication during the data collection period. Consequently, the findings derived from our study may not be readily generalizable to individuals or groups falling into these categories.

Furthermore, numerous studies have highlighted racial disparities in pain perception, assessment, and treatment across diverse chronic pain conditions. For example, African American patients, in comparison to Caucasian Americans, tend to report higher pain severity and pain-related functional disability. Also, African American patients are less likely to seek care for back pain, even when controlling for other demographic factors (Shekelle et al., 1995). The demographic of our survey respondents indicates a predominant representation of Caucasian Americans (76%). This raises concerns regarding the generalizability of our findings to CLBP patients from diverse racial backgrounds or other minoritized populations.

### Future directions

Considering the correlational nature of the present study, longitudinal or experimental studies are warranted to establish directional associations between psychosocial factors and CLBP symptoms, as only through such research can we unravel how these variables are causally related. Moreover, since our findings are derived from a sample of CLBP patients undergoing pharmacological treatment, researchers should also consider collecting data from patients who (1) do not seek medical treatment, (2) employ different types of medical treatments for CLBP (e.g., over-the-counter [OTC] medications, acupuncture, massage, spine surgery techniques), or have pain caused by physical injuries. Researchers may also collect information from the caregivers and/or significant others of patients.

Notably, our sample consisted mostly of Caucasian Americans. Therefore, it is important to acknowledge that our findings may not be applicable to CLBP patients from various racial or cultural backgrounds. Accordingly, future studies may further expand knowledge of CLBP by collecting samples from diverse minoritized groups. Lastly, future research may improve our understanding of CLBP by investigating the interactions between various psychosocial and demographical characteristics to further explain symptoms and experiences related to CLBP. Extant pain literature has suggested that males tend to use more avoidant coping strategies such as distraction, denial of pain, and suppressing their expressions of pain, while females are often encouraged to express their pain-related worries as an attempt to cope with pain and seek social support (Berkley, 1997; Myers et al., 2001; Diotaiuti et al., 2022). Incorporating demographic attributes into our integrated approach to CLBP may shed light on personalized interventions/treatments that address the needs and difficulties experienced by various groups of patients as they cope with CLBP.

## Conclusion

Though our study is not the first to examine the role of attachment insecurity in CLBP patients’ pain experiences, we extended previous work on CLBP by examining interpersonal factors, such as anxious attachment and avoidant attachment, while taking intrapersonal ER strategies into account. Our findings suggest that anxious attachment, emotional expression, and expressive suppression may be risk factors associated with CLBP severity and interference. Additionally, emotional expression may be associated with more pain severity and interference among people with low and medium levels of avoidant attachment. We hope that our findings motivate CLBP researchers and clinicians to integrate both interpersonal and intrapersonal perspectives to understand the pain experience of CLBP patients more comprehensively.

## Data availability statement

The raw data supporting the conclusions of this article will be made available by the authors, without undue reservation.

## Ethics statement

The studies involving humans were approved by Office of Research Compliance, Ohio University. The studies were conducted in accordance with the local legislation and institutional requirements. The ethics committee/institutional review board waived the requirement of written informed consent for participation from the participants or the participants’ legal guardians/next of kin because this study was conducted online where written informed consent was not available to obtain.

## Author contributions

YY: Conceptualization, Data curation, Formal analysis, Investigation, Methodology, Project administration, Writing – original draft. DM: Conceptualization, Funding acquisition, Methodology, Project administration, Resources, Supervision, Validation, Writing – review & editing.
